# Muscle Fibre Architecture of Thoracic and Lumbar Longissimus Dorsi Muscle in the Horse

**DOI:** 10.3390/ani11030915

**Published:** 2021-03-23

**Authors:** Johanna Dietrich, Stephan Handschuh, Robert Steidl, Alexandra Böhler, Gerhard Forstenpointner, Monika Egerbacher, Christian Peham, Hanna Schöpper

**Affiliations:** 1 Institute of Anatomy, Histology and Embryology, Department of Pathobiology, University of Veterinary Medicine, 1210 Vienna, Austria; johannadietrich@gmx.at (J.D.); gerhard.forstenpointner@vetmeduni.ac.at (G.F.); monika.egerbacher@gmail.com (M.E.); h.schoepper@uni-bonn.de (H.S.); 2 Movement Science Group Vienna, Equine Clinic, Department for Companion Animals and Horses, University of Veterinary Medicine, 1210 Vienna, Austria; robert.steidl@gmail.com; 3 VetImaging, VetCore Facility for Research, University of Veterinary Medicine, 1210 Vienna, Austria; Stephan.handschuh@vetmeduni.ac.at; 4 Diagnostic Imaging, Department for Companion Animals and Horses, University of Veterinary Medicine, 1210 Vienna, Austria; alexandra.boehler@gmx.net

**Keywords:** erector spinae, fascicle length, pennation angle, skeletal muscle, horse

## Abstract

**Simple Summary:**

As the longissimus dorsi muscle is the largest muscle in the equine back, it has great influence on the stability of the spine and facilitates proper locomotion. In general, muscle function is determined by its specific intramuscular architecture. However, only limited three-dimensional metrical data are available for the inner organisation of the equine longissimus dorsi muscle. The thoracic and lumbar longissimus muscles of five formalin-fixed cadaveric horse backs of different ages and body types were dissected layerwise from cranial to caudal. Three-dimensional coordinates along individual muscle fibre bundles were digitised from the origin to the insertion and 3D models were created using imaging software and computed tomography. The muscle was divided into functional compartments and morphometric parameters (muscle fascicle length, pennation angles, muscle volume and the physiological cross-sectional area (PCSA)) were determined. Fascicle length showed the highest values in the thoracic region and decreased from cranial to caudal, while in most caudal compartments, fascicle length was less than 50% of the fascicle length in thoracic compartments. The pennation angles differ between compartments. In the cranial compartment, fascicles almost run parallel to the horizontal plane (mean angle 0°), while in the caudal compartment, the angles increase up to a mean angle of 38°. In the sagittal plane, the pennation angles varied from parallel (0°) in cranial compartments to 0–22° in the caudal compartments. The muscle volume ranged from 1350 cm^3^ to 4700 cm^3^ and PCSA from 219 cm^2^ to 700 cm^2^. This study lays the anatomical basis for a biomechanical model to simulate muscle function.

**Abstract:**

As the longissimus dorsi muscle is the largest muscle in the equine back, it has great influence on the stability of the spine and facilitates proper locomotion. The longissimus muscle provides support to the saddle and rider and thereby influences performance in the horse. Muscular dysfunction has been associated with back disorders and decline of performance. In general, muscle function is determined by its specific intramuscular architecture. However, only limited three-dimensional metrical data are available for the inner organisation of the equine longissimus dorsi muscle. Therefore, we aimed at investigating the inner architecure of the equine longissimus. The thoracic and lumbar longissimus muscles of five formalin-fixed cadaveric horse backs of different ages and body types were dissected layerwise from cranial to caudal. Three-dimensional coordinates along individual muscle fibre bundles were recorded using a digitisation tool (MicroScribe^®^), to capture their origin, insertion and general orientation. Together with skeletal data from computed tomography (CT) scans, 3D models were created using imaging software (Amira). For further analysis, the muscle was divided into functional compartments during preparation and morphometric parameters, such as the muscle fascicle length, pennation angles to the sagittal and horizontal planes, muscle volume and the physiological cross-sectional area (PCSA), were determined. Fascicle length showed the highest values in the thoracic region and decreased from cranial to caudal, with the cranial lumbar compartment showing about 75% of cranial fascicle length, while in most caudal compartments, fascicle length was less than 50% of the fascicle length in thoracic compartments. The pennation angles to the horizontal plane show that there are differences between compartments. In most cranial compartments, fascicles almost run parallel to the horizontal plane (mean angle 0°), while in the caudal compartment, the angles increase up to a mean angle of 38°. Pennation angles to the sagittal plane varied not only between compartments but also within compartments. While in the thoracic compartments, the fascicles run nearly parallel to the spine, in the caudal compartments, the mean angles range from 0–22°. The muscle volume ranged from 1350 cm^3^ to 4700 cm^3^ depending on body size. The PCSA ranged from 219 cm^2^ to 700 cm^2^ depending on the muscle volume and mean fascicle length. In addition to predictable individual differences in size parameters, there are obvious systemic differences within the muscle architecture along the longissimus muscle which may affect its contraction behaviour. The obtained muscle data lay the anatomical basis for a specific biomechanical model of the longissimus muscle, to simulate muscle function under varying conditions and in comparison to other species.

## 1. Introduction

Back pain is a very common problem in horses and very hard to detect. It is often discovered too late by poor performance or gait abnormalities [1]. Due to the variety of possible clinical signs, equine back problems are often challenging to diagnose [2,3,4]. To avoid long-term effects, such as secondary skeletal modifications, early detection of pain and its cause is essential and poses a challenge to horse owners and veterinarians [5,6].

The longissimus dorsi muscle (LG) is the largest muscle of the back and is part of the active epaxial musculoskeletal system, providing stability of the spine [7,8]. 

A decrease in spinal stability and muscle strain of the epaxial back muscles are considered the main causes of back pain in humans [9]. Because of the important role of the thoracolumbar longissimus dorsi muscle, especially in the ridden horse [8], it is plausible that strain in this muscle might lead to back pain.

Skeletal muscle architecture describes the internal arrangement of the muscle fibres in a muscle and partly determines the contraction behaviour [10]. Bundles of muscle fibres are surrounded by perimysium—a sheath of connective tissue. Those muscle fibre bundles or fascicles are distinguishable in formalin-fixed tissue and can therefore be used to characterise muscle architecture. Architectural parameters, such as fascicle length and pennation angle, have a major impact on the contractibility and muscle function [11,12,13,14,15]. Therefore, muscle morphology clearly determines muscle function.

Besides architectural parameters, metabolic parameters, such as muscle fibre type distribution, also influence muscle function [12,14]. Previous anatomical studies of the equine back mainly focus on the general skeletal and ligamentous structures and their function [16,17,18], whereas detailed data and knowledge about the inner muscle architecture are limited. Von Scheven (2001) and Ritruechai et al. (2008) gave important insight into anatomical characteristics of the equine longissimus muscle. Ritruechai et al. (2008) showed segmental variations in the cross-sectional area and moment arm length, as well as differences in direction cosines measured in a multitude of segments. This work unravelled regional specialisation of the longissimus dorsi muscle, also suggesting functional consequences of the anatomical characteristics. However, the segmental methodological approach and evaluation of mean direction cosines left scope for interpretation. We therefore undertook the challenge to reduce intervals of measurement, increase resolution and quantify fascicle length and three-dimensional orientation. In addition, we aimed at producing a 3D model of the completely thoracolumbar LG in order to visualise inner muscle structure of the largest muscle of the horse back and lay a necessary quantitative basis for future biomechanical models that could be used to mimic or simulate muscle function.

Additional information, like PCSA, total length and volume, should give insight into the general longissimus muscle architecture and support already available literature. 

With the available literature, the following hypotheses are formulated: fascicle length will decrease and PCSA will increase from cranial to caudal aspects of the LG. In addition, the fascicle orientation to the spine will change along the length of the LG. In addition to the description and measurements of inner muscle architecture of the LG as one of the most important back muscles, a three-dimensional visualisation will help understand muscle structure more thoroughly and also be of great didactic value for veterinary anatomy training.

## 2. Material and Methods

### 2.1. Specimen Preparation

In this study, cadaveric backs obtained from five horses from the anatomical collection of the University of Veterinary Medicine Vienna were used to examine the thoracic and lumbar LG. None of the horses had any known musculoskeletal problems. All animals were euthanised or died at the equine hospital of the University of Veterinary Medicine Vienna, for reasons unrelated to this study. As the study was performed on dead horses, it did not require ethical approval. However, the animals owners’ consent to dissect and publish resulting data was obtained according to the standard procedure, which was approved by the ethics and animal welfare committee of the University of Veterinary Medicine Vienna. The horses were dissected according to the “Good Scientific Practice and Ethics in Science and Research” regulation emplemented at the University of Veterinary Medicine Vienna. 

Only small breeds, ponies of different breeds and a Standardbred foal (one Shetland pony, one Shetland–Haflinger crossbreed, one Dutch Riding Pony, one Icelandic Horse and one Standardbred) were used, as the storage space as well as computed tomography (CT) gantry dimensions were limited. The age ranged between two months and 29 years. The body mass ranged from 119 kg to 280 kg with mean ± SD of 177.2 kg ± 58.02. The horses were for recreational use.

Prior to LG preparation, skin, cutaneous muscle, fat and muscles of the shoulder girdle, such as the latissimus dorsi, dorsal and ventral serratus muscle and trapezius muscle as well as other epaxial back muscles like the spinalis muscle and the gluteus medius muscle, as an intrinsic muscle of the hindlimb, were removed, in order to expose the region of interest. The backs were trimmed to a minimal size, ranging from the 6th cervical vertebra to the pelvis. Subsequently, they were fixed in 4% formalin solution to preserve the tissue.

To guarantee an adequate fixation of the muscle, formalin was also injected into the multifidi muscles and into the iliocostalis muscle along the thoracolumbar LG. The LG thoracis et lumborum itself was not injected with fluid to avoid local muscle fascicle compression. Then the whole backs were stored in a formalin tank until further processing.

### 2.2. Dissection, Digitisation, Three-Dimensional Modelling

Screws were drilled into the spinous processes (from the 6th cervical vertebra to the sacrum), the coxal tuber and the body of the ribs to serve as reference coordinates during the digitisation process. Helical CT scans of the formalin-fixed backs were performed with a 16-slice helical CT scanner (Somatom Emotion, Siemens AG Medical Solutions, Erlangen, Germany) from the last cervical vertebrae to the pelvis including the os sacrum. Technical settings used for the scans were 130 kV tube voltage, 150–200 mAs tube current, collimation of 1.2 or 0.6 in one case, respectively, rotation time of 1–1.5 s and slice thickness of 0.75–1.5 mm. The CT DICOM image sequences were imported into the 3D software package Amira (version 5.3.3, Visage Imaging, Berlin, Germany) and a surface model of each skeleton was created. All skeleton models were oriented into a standardised coordinate system to make the obtained data comparable. 

**Figure 1:**
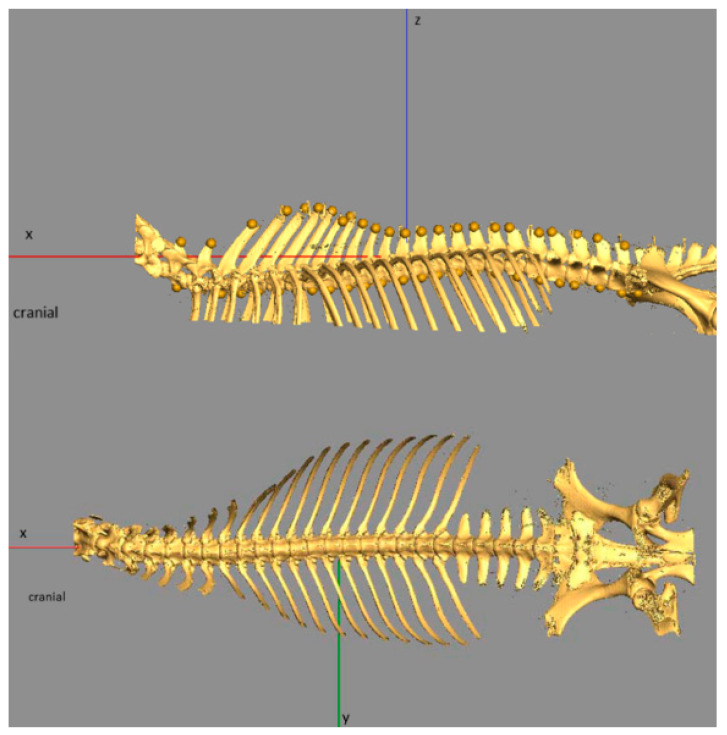
Dorsal and left lateral view of the rendered three-dimensional skeleton model of a horse back. Neck region removed cranial to the 6th vertebra, thoracic and lumbar ribs reduced to dorsal aspects, bones of the pelvic limbs removed distal to the hip bone and caudal bones removed. Models of the skeleton were aligned according to a standardised reference coordinate system as described above. The *x*-axis was defined as cranio-caudal axis, the *y*-axis as left–right axis and the *z*-axis as dorsoventral axis.

For establishing this standardised coordinate system, we used a set of reference points dorsal to the spinous processes and ventral on the ventral crest of the body of each thoracic and lumbar vertebra. From this point cloud, the three principal axes were calculated. The *x*-axis was defined as the cranio-caudal axis, the *y*-axis as the left–right axis and the *z*-axis as the dorsoventral axis (see Figure 1).

The thoracic and lumbar part of the LG was dissected in layers from cranial to caudal and superficial to deep. Layer thickness was adjusted to the total muscle thickness and degree of variance of muscle fascicle orientation (approx. 2–20 mm). The thinner the muscle and the more changes were seen in fascicle orientation, the thinner the layers were designed. For each layer, three-dimensional coordinates of representative muscle fascicle were captured, using a Microscribe G2X digitiser (Revware Systems, Inc., San Jose, CA, USA). At least three fascicles were recorded per layer. Origin, insertion and at least three points in between along the course of the fascicles were recorded. Muscle fascicles are bundles of muscle fibres that are surrounded by a sheath of connective tissue—the perimysium. Those muscle fibre bundles or fascicles are distinguishable in formalin-fixed tissue and could therefore be used to characterise muscle architecture.

This procedure was repeated layer by layer for the whole muscle length and for both body sides, in order to display and describe the inner fascicle structure for the entire thoracolumbar LG.

For each fascicle digitisation session, reference points in neighbouring spinous processes, ribs and coxal tuber were also digitised and with their help the digitised muscle data could be imported to Amira and aligned to the surface model of the same skeleton within the same reference system, as described before.

### 2.3. Data Analysis 

**Figure 2:**
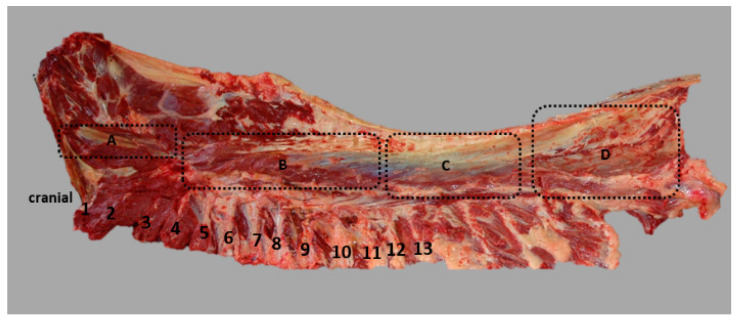
Left lateral view of a freshly dissected horse back. Neck region removed cranial to the 6th vertebra, thoracic and abdominal wall reduced to most dorsal aspects, hind limbs exarticulated and tail removed. The thoracolumbar longissimus dorsi muscle (LG) is exposed after removing skin, fat and overlying muscles. Ribs are numbered cranial to caudal (numbers 1–13). The thoracolumbar LG was divided macroscopically into compartments **A**–**D**. Compartment **A** describes the most cranial part of the muscle inserted at the last cervical vertebrae, followed by compartment **B** which overlaps compartment **A** in the cranial part at the height of the 4th rib. The caudal part of compartment B reaches to the height of the 13th rib. Compartment **C** comes right behind compartment **B** and reaches from the height of the 13th rib to the impression of the removed Musculus gluteus medius. Compartment **C** is followed by compartment **D**, which is the most caudal part of the muscle, becoming visible after removing the gluteus medius muscle. Compartment **D** is the most caudal part of the muscle that is in close contact with the gluteus medius muscle, as it inserts at the aponeurosis of the LG. It has a slim dorsal muscle belly that originates at the dorsal processes of the sacrum and a ventral flat portion, which runs from the iliac crest.

For further description and analyses, the muscle was divided macroscopically into four compartments (**A**–**D**, see Figure 2), with compartment **A** being the most cranial part inserted at the last cervical vertebra, followed caudally by compartment **B** and **C**. 

**Figure 3:**
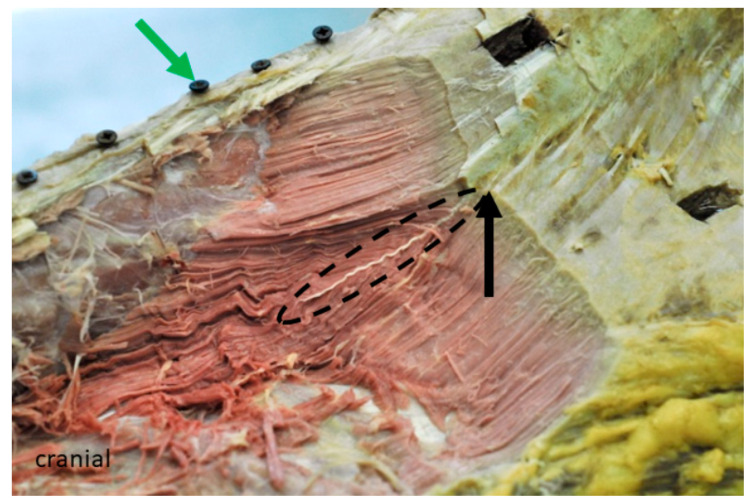
Left cranio-lateral view of a formalin-fixed horseback, showing the partly dissected lumbar LG at the height of the 16th rib (compartment **C**). The green arrow is pointing at a screw drilled into the spinous process of the 15th thoracic vertebra, serving as a reference point during computed tomography (CT) scan and digitisation process. Muscle layers have been ablated from cranial to caudal to expose MFB from their origin to the insertion. The strong aponeurosis releases a fascia (black arrow) that divides the muscle partly into a supra- and subfascial belly.

**Figure 4:**
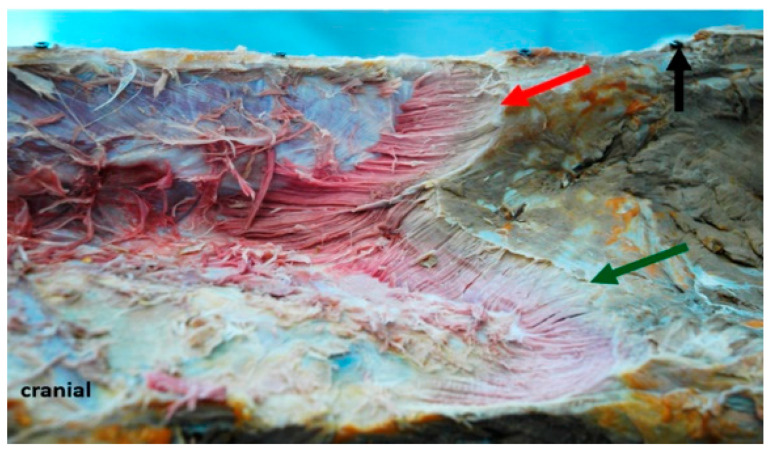
Left cranio-lateral view of a formalin-fixed horseback, showing the partly dissected lumbar LG in compartment **D**. The red arrow is pointing at the slim dorsal muscle belly that originates at the dorsal processes of the sacrum. The green arrow points at the flat ventral muscle portion running from the iliac crest. Muscle layers have been removed from cranial to caudal to expose fascicles from their origin to the insertion. The screw that is drilled into the dorsal process of the sacrum (black arrow) served as a reference point.

**Figure 5:**
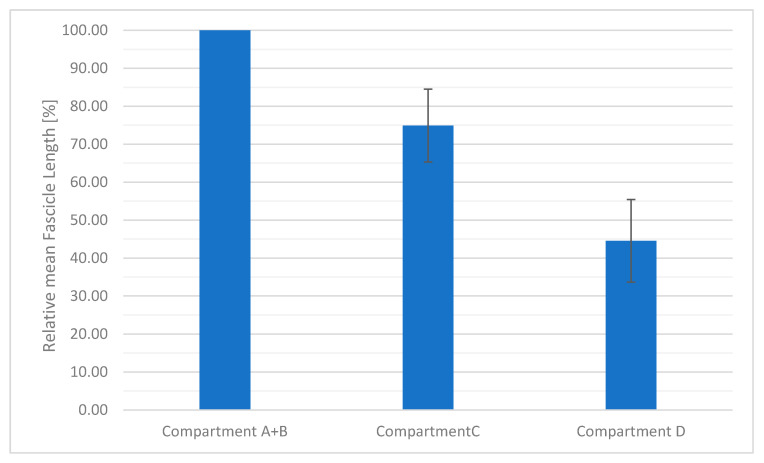
This diagram shows the relative mean fascicle lengths (%) of all animals for cranial (compartment **A** + **B**), middle (compartment **C**) and caudal (compartment **D**) of the LG. Fascicle lengths decrease from cranial to caudal. Fascicle length of compartment **A** + **B** (longest fascicle length) was set to 100%.

Architectural parameters such as fascicle length, muscle volume, physiological cross-sectional area (PCSA) and pennation angles were calculated from digitised data or obtained from the axial CT scans of the backs. The fascicle length was calculated with MATLAB R2015b (The MathWorks, Inc., Natick, MA, USA) as the sum of the distances between the consecutive digitised points between origin and insertion of each digitised fascicle. Mean fascicle length was calculated for each compartment of the LG (see Figures 3–5). To compare between animals of different sizes, relative fascicle lengths were used. One hundred percent was set for the compartments with the longest mean fascicle lengths. As in compartment **A** and **B**, due to the thin shape of the muscle, fewer data for fascicle length could be collected, and data for these two compartments, **A** and **B**, were pooled for fascicle length calculations. 

Muscle volume was determined for the right longissimus muscle of all animals with the help of the CT image volumes based on manual segmentation in Amira (version 5.3.3, Visage Imaging, Berlin, Germany). As no specific side differences were assumed, we only used the right side data for determining muscle volume. Due to tissue shrinkage that occurs during the fixing process in formalin, the overall measurements are slightly shorter than the in vivo situation [19]. Therefore, we do not discuss quantitative but relative data that allow for seeing the biologically relevant differences across intra- and inter-individual longissimus muscle.

Finally, PCSA was calculated for the LG by dividing the muscle volume by the mean fascicle length of the muscle.

**Figure 6:**
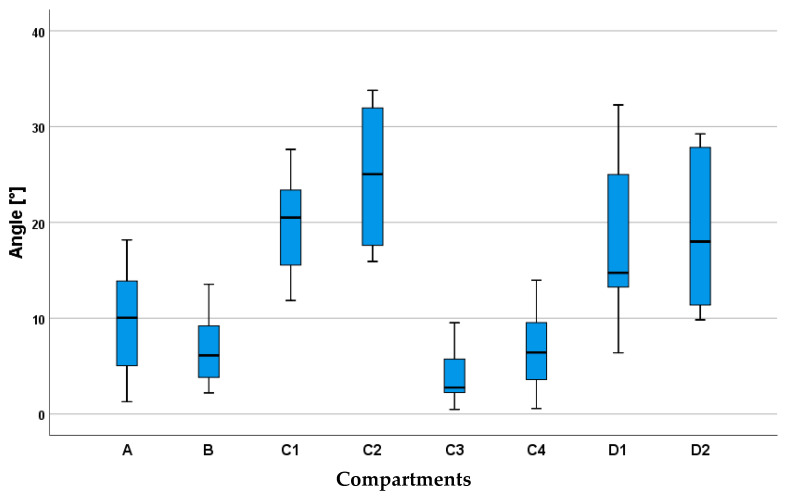
Pennation angles to the sagittal plane calculated for each compartment **A**–**D** and sub-compartment **C1**–**C4** and **D1** and **D2**. Angles above zero degrees describe fascicle orientation in a medial direction to the spine. The boxplot or box and whisker plot depicts the quartiles (2 quartiles in the box and 2 out of the box indicated by the whiskers) and the median (bold line in the box) of the distribution.

To describe the orientation of the fibre bundles, pennation angles were measured as 2D projected angles between the fascicles (assumed as straight lines from origin (first point of digitalisation) to insertion (last point of digitalisation)) and anatomical planes of the reference coordinate system. Two angles were determined for fibres of each compartment: (i) the horizontal projection of the angle between fascicle and sagittal plane (xz plane) and (ii) the sagittal projection of the angle between fascicle and horizontal plane (xy plane). Data are given as boxplots (see Figures 6 and 8) and mean + standard deviation (SD) angles. Digitised fascicle orientation enabled us to generate 3D angles (visualised in the 3D model)—however, we decided to present 2D values for the pennation angles in two different planes as they are much easier to understand and more suitable in the anatomical description. 

The collected data were processed in Microsoft Excel 2010 (Microsoft Corporation, Redmond, WA) and calculated using MatLab R2015b (The MathWorks, Inc., Natick, MA, USA). Data are given as mean values with standard deviation. A linear regression (goodness of fit (R^2^)) was calculated to show that the fascicle length as well as the orientation (pennation angles) of the fascicle change from cranial to caudal.

## 3. Results

### 3.1. Anatomical Description 

The thoracolumbar LG extends in its total expansion from the sacrum and iliac wing all along the vertebral column to the last cervical (C7) vertebra. Along its length, the LG inserts into the mammillary, transversal and dorsal spinous processes and to the ribs. It has its greatest dimension in the lumbar region while it narrows from caudal to cranial. The muscle is covered by the thoracolumbar fascia that releases an inner fascia at the height of the 13th rib, which partly divides the muscle. The inner muscle architecture varies widely along the length of the LG. Therefore, different compartments were defined and described from cranial to caudal, shown in detail below.

Compartment **A** is the most cranial part of the muscle. Here, the muscle inserts into the transversal process of the last cervical vertebra via flat tendons. The muscle strand is thin and fascicles run almost parallel to the spinal column. Most fascicles are long and span over several vertebral segments. In addition, shorter fascicles can be found, particularly in the deeper layers of the muscle. The caudal part of compartment **A** is overlapped by the muscle belly of the following compartment **B** and only fully visible after removing cranial parts of compartment **B**, see Figure 2.

At the height of the 5th rib, the cranial part of compartment **B** follows and partly overlaps compartment **A**. The muscle becomes bulkier and muscle diameter increases. The fascicles run almost parallel to each other in a caudodorsal to cranioventral direction. Macroscopically, fascicles are shorter than in compartment **A**. The aponeurosis of the muscle provides origin for the fascicles that run in a uniform course parallel to each other towards their insertion at the articular, mammillary and transversal processes of the thoracic vertebrae.

The diameter of the muscle continues to increase from the height of 13th to 18th rib, where the muscle has its largest expansion. Compartment **A** and **B** were joined as one functional unit. They were only split during the preparation process, because of some overlapping muscle parts, and then rejoined in the analysis.

The separation between compartment **B** and **C** is not apparent from the outside of the muscle, because of the thoracolumbar fascia that covers the muscle. In the lumbar region, the fascia gets thicker and joins the supraspinous ligament. The classification is based on the internal structure of the muscle, as at the height of the 13th rib, the thoracolumbar fascia releases a fascia sheet, which divides approximately half of the muscle into a dorsomedial and a ventrolateral portion. 

The thoracolumbar fascia provides the origin for fascicles that span between the fascia and the ribs, as well as between the fascia and the aponeurosis of the multifidus muscle. Compartment **C** shows the structure of a multipennate muscle, as the inner fascia not only divides the muscle into two parts, but also provides the origin for fascicles that span between the inner fascia and the deep layer of the fascia spinocostotransversalis. In compartment **C**, fascicles are no longer parallel to each other, as the inner fascia as the origin influences the orientation of the fascicles. To describe and discuss the results, the fascicles are grouped according their orientation and therefore also their origin. Fascicles lying dorsal to the inner fascia and arising at the thoracolumbar fascia are grouped in compartment **C1**. Fascicles lying dorsally to the inner fascia and originate at the inner fascia are assigned to **C2**. In compartments **C1** and **C2**, fascicles are orientated from caudolateral to craniomedial. 

Fibres that are situated ventrally to the inner fascia are divided into compartment **C3** and **C4**. Muscle fascicles that originate at the inner fascia are grouped in **C3** and fascicles that span between the thoracolumbar fascia and the ribs, having no contact with the inner fascia, are assigned to **C4**. In compartments **C3** and **C4**, fascicles are orientated from caudodorsal to cranioventral, see Figure 3.

After removal of the gluteus medius muscle that attaches to the aponeurosis of the LG, as far forward as the 18th thoracic vertebra, compartment **D** becomes visible. In this region, the LG can be divided macroscopically into two compartments. It has a dorsal thin muscle belly, which reaches the os sacrum and a ventral wide and flat portion that attaches to the iliac crest. Fascicles from the dorsal portion are grouped in compartment **D1**, fascicles from the flat ventral portion are assigned to compartment **D2**. Fascicles in compartment **D1** originate at the thoracolumbar fascia and insert at the mammillary, transversal and dorsal spinous processes. They also attach to the fascia that covers the multifidi muscles and are orientated from caudolateral to craniomedial, while in compartment **D2**, fascicles run from caudodorsal to cranioventral, as they originate at the thoracolumbar fascia and attach to the ribs, see Figure 4.

### 3.2. Inner Muscle Architecture

All data are presented as mean ± SD. Mean fascicle length of all animals was 65.09 ± 24.71 mm with a minimum length of 12.47 mm and a maximum length of 202.19 mm. Fascicle length showed the highest values in the cranial compartments (**A** + **B**) and decreased from cranial to caudal. Compartment **A** + **B** with the longest fascicle length was set to 100%. The middle part of muscle compartment **C** showed 74.93 ± 9.6% compared to the fascicle length in compartment **A** + **B**. In compartment **D**, fascicle length was less than half of the length (44.53 ± 10.86%) of the fibre bundle length in compartment **A** + **B** (see Figure 5).

The muscle volume of the LG ranged between 1350 cm^3^ and 4700 cm^3^ with a mean (±SD) volume of 2180 ± 1269.88 cm^3^. The PCSA was calculated for each examined longissimus muscle and ranged, depending on the muscle volume and mean fascicle length, from 219.42 cm^3^ to 700.40 cm^3^ with a mean PCSA of 365.53 ± 174.07 cm^3^.

**Figure 7:**
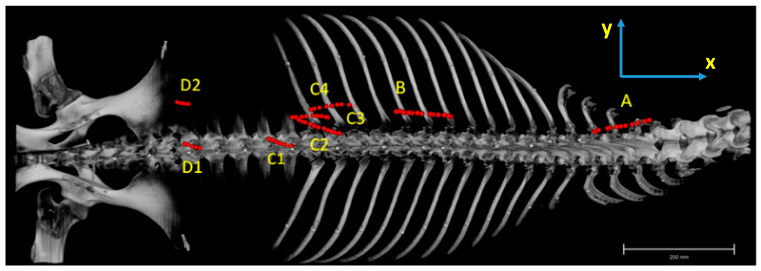
Dorsal view of a rendered CT bone model of a horseback. Red lines demonstrate the orientation (to the sagittal plane) of one representative fascicle for each compartment and sub-compartment (yellow letters) for the left longissimus dorsi muscle.

Horizontally projected pennation angles of the fascicles relative to the sagittal plane (describing the orientation of the fascicles in the xy relative to the xz plane—see Figure 1) ranged from 0.5 degrees to 34 degrees and varied between compartments. An angulation of 0 degrees means that the fascicle runs parallel to the sagittal plane (spine). For the left body side, negative angles indicate that the fascicles are running from medial (origin) to more lateral (insertion), while positive angles indicate that fascicles are running from lateral (origin) to more medial (insertion). In compartment **A**, the mean angle is 8.8 ± 6.0°, with the fascicles running almost parallel to the spine and slightly in the lateral direction. Fascicles in compartment **B** almost run parallel to the spine with a mean angle of 8.8 ± 5.9°. In compartment **C1**, the direction of the fascicle is influenced by the inner fascia and therefore the pennation angles varied widely within this muscle part. For the fascicles lying dorsally to the fascia, the mean angle was 20.1 ± 4.9° and for the fascicles that arise at the fascia **C2**, the mean angle was 24.3 ± 6.6°, while the mean angle for the ventrally lying fascicles in **C3** was 3.6 ± 2.7° for the fascicles arising at the fascia **C4** and 6.7 ± 3.8° for the ventral ones. In compartment **D**, the mean angle was 16.6 ± 9.1° and varied between 4.9° and 32.3° for fascicles of the dorsal muscle part (**D1**) and 19.1 ± 7.7° for the ventral fascicles (**D2**), with sub-compartment **D2** showing most mean variation, see Figure 6 and Figure 7.

**Figure 8:**
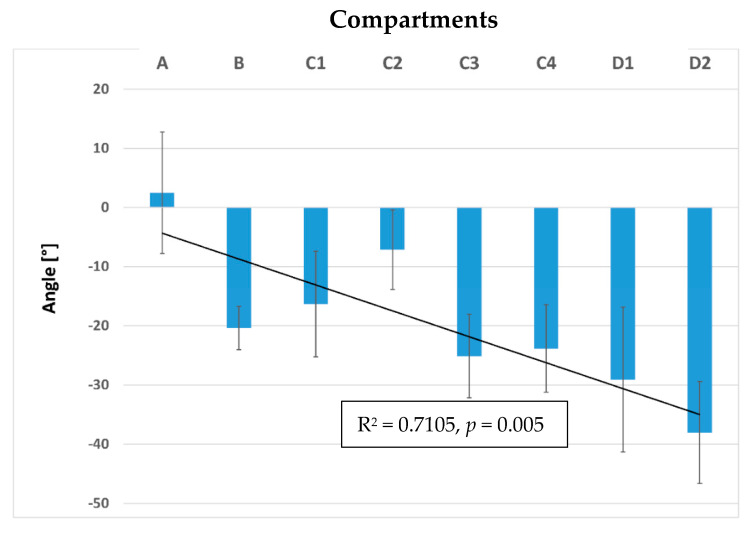
Mean pennation angles of the longissimus dorsi muscle to the horizontal plane calculated for each compartment **A**–**D** and sub-compartments **C1**–**C4** and **D1** and **D2**. Compared to cranial compartment **A**, the pennation angles to the horizontal plane change the orientation and get steeper in the caudal direction (see significant linear regression, R^2^ = 0.71, *p* = 0.005), with the lowest values found in compartment **D**.

**Figure 9:**
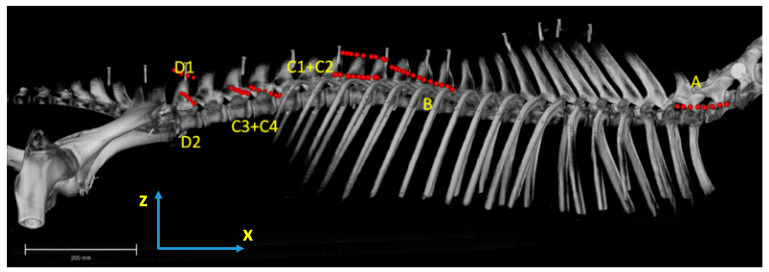
Right lateral view of a rendered CT bone model of a horse back. For each compartment and sub- compartment (indicated with the yellow letters) for the longissimus dorsi muscle, the orientation of one representative fascicle to the horizontal plane is shown in red.

Sagittaly projected pennation angles of the fascicles relative to the horizontal plane describe the orientation of the fascicles in the xz relative to the xy plane (see Figure 1). An angle of zero degrees means that a fibre is parallel to the horizontal plane, while positive angles indicate a fibre course from caudodorsal (origin) to cranioventral (insertion). Negative angle values would indicate fibre course from ventral (origin) to dorsal (insertion), but did not occur in the present study. In the cranial part of the muscle (compartment **A**), the fascicles run almost parallel relative to the horizontal plane (xy plane), and the mean angle was 2.5 ± 10.3°. The more caudal the fascicles were situated, the steeper the angles. While in compartment **B** the mean angle was −20.4 ± 3.6°, in compartment **C**, the mean angle varied from fascicles dorsally to the inner fascia between **C1** (−16.3 ± 8.9°), and **C2** (−7.2 ± 6.7°). In **C3** and **C4**, the angle was reduced and lay between −25.1 ± 7.0° for **C3** and −23.8 ± 7.4° for **C4**. In the most caudal part of the muscle compartment **D**, the angles to the horizontal plane showed a mean angle of −29.1 ± 12.2° in the dorsal part (**D1**) and −38.0 ± 8.6° in the ventral part (**D2**), see Figure 8 and Figure 9.

## 4. Discussion

Muscle performance highly depends on architectural parameters such as fascicle length and pennation angle. Thus, a detailed knowledge of inner muscle architecture is necessary to understand muscle function during movement [12,14,20,21,22]. Using dissection and digitisation in five horse backs, we were able to capture architectural data of the thoracolumbar LG. With the help of three-dimensional reconstructions, regional differences in fascicle architecture of the thoracolumbar LG of the horse could be quantified and documented for the first time.

### Muscle Architecture 

The LG muscle is considered an epaxial muscle with a segmental structure. Here, we give a detailed anatomical description of the anatomical subdivisions of the equine thoracolumbar LG and compare them in terms of muscle architecture parameters. While compartment **A** and **B** are both unipennate, **A** inserts at the last cervical vertebra and compartment **B** at the first thoracic vertebrae and ribs. In compartment **C**, an internal fascia is apparent leading to a completely different arrangement of fascicles and bipennate inner structure. The most caudal portion of the lumbar LG, compartment **D**, can be divided into an upper belly reaching the os sacrum, while another rather flat portion is attached to the iliac crest. Whether a muscle is unipennate or mulitpennate also influences fascicle length and contractile forces. Fascicle length showed the highest values in the cranial compartment (**A** + **B**) and decreased from cranial to caudal, with compartment C showing 75% of the cranial fascicle length, while in compartment **D**, fascicle length was 45% of the fascicle length in compartment **A** + **B**. Therefore, we can conclude that fascicle length decreases from cranial to caudal.

Our results show that the fascicle length decreased from cranial to caudal, with most caudal fibres in compartment **D** being about 40% of the length of the fascicles in the cranial–thoracic longissimus muscle, therefore, this part of the hypothesis is supported. This is in accordance with previous studies [8,23] that also reported that the muscle fascicles get shorter from cranial to caudal. The high standard deviation is a result of the different horses used for this study. Fascicle length depends on how many sarcomeres are arranged in series. Longer fascicles have a greater contraction velocity and a greater excursion over which a muscle can exert its force [24,25,26]. The longer fascicles of the thoracic part of the LG indicate that the LG has a greater muscle excursion and contraction velocity in that region compared to the lumbar region, where fascicles are shorter. Studies about the mobility of the spine in the horse showed that the movement between the vertebrae decreases from cranial to caudal due to differences in intervertebral articulation. Between the second and the 17th thoracic vertebra, most lateral flexion occurs, while there is almost no dorsoventral flexion. The spine between the 17th thoracic and the sixth lumbar vertebra shows the least mobility, while between the sixth lumbar vertebra and the os sacrum, a lot of dorsoventral flexion occurs [27]. The greater range of thoracolumbar mobility between the 14th and 18th thoracic vertebra [28] predisposes the thoracic LG with its long muscle fascicles to play an important role in movement and lateral bending of the spine.

The pennation angles to the horizontal and sagittal plane become bigger in the lumbar region, compared to the thoracic region. In the thoracic part of the longissimus, fascicles run almost parallel to the spine (sagittal plane) and to the horizontal plane, while in the lumbar region (compartment **C** + **D**), the angles get steeper and the fascicles no longer run parallel to each other, as the inner fascia influences the orientation of the fascicles The last part of the hypothesis (the orientation of the fascicle to the spine will change along the length of the LG) can be supported.

Other studies on the equine LG [23] calculate the pennation angle as the angle between the aponeurosis or internal fascia and the muscle fascicle. 

We calculated the pennation angles to the horizontal and sagittal plane to describe the orientation of the fascicles [15]. The pennation angles become larger from cranial to caudal. 

The pennation angles of the cranial thoracic LG (compartment **A** + **B**) indicate a high contraction velocity and potential for high lateral excursions. Together with the fact that the fascicles almost run parallel to the spine, this suggests that the thoracic longissimus muscle rather plays a role in motion rather than in stabilisation, especially as a lateral flexor of the spine [23,29].

In contrast, in pennate muscles, more muscle fibres can be packed into the muscle volume. Therefore, higher forces can be generated [14,30]. In the lumbar region of the LG, the inner muscle architecture changes compared to the more cranial compartments. While the fascicle length decreases from cranial to caudal, the fascicles no longer run parallel to the horizontal and sagittal plane. The lumbar LG is partly divided by the inner fascia into a dorsomedial and a ventrolateral portion. The fascia not only divides the muscle, it also provides an insertion origin for fascicles that are attached to it. 

This makes the muscle be built up like a multipennate muscle. The inner structure of the longissimus in the lumbar region (compartment **C**, **D**) indicates that in this region, the muscle can exert large forces and plays an important role in stabilisation of the spine. Our results are in line with previous studies [8,23], which found that the lumbar LG has the potential to excert greater forces than the thoracic LG, due to its inner architecture.

These architectural findings match with kinematic studies [28,31,32] that showed a higher proportion of excursion in the cranial thoracic vertebral column. The thoracic part of the LG has long fascicles that almost run parallel to the vertebral column, which results in a high contraction velocity and the prevalence of a larger excursion, as mentioned before.

Stability of the thoracic vertebral column is provided by the rib cage and other trunk muscles. In the lumbar region, the LG has the largest PCSA. In addition to the large PCSA in the lumbar region, short fascicle lengths and big pennation angles underline the important role in stabilisation of the spine in this area. 

In the caudal part, the LG stays via its aponeurosis in close contact with the middle part of the gluteal muscle that is one of the major propulsion muscles of the hindlimb. In this area, the muscle seems to have an important role in stabilising the lumbar spine against dynamic forces during push-offs of the hind limbs [16]. In pennate muscles, as the caudal compartments of the LG, fascicles rotate to greater angles of pennation as they shorten [33]. For that reason, the velocity of the contracting fibres can be slower compared to the muscle velocity and the caudal compartments of the LG might still be capable of fast contraction.

Using muscle volume and fascicle length, the PCSA can be estimated. It describes the number of parallel sarcomeres and is directly proportional to the force a muscle can exert [34,35]. It is therefore a good parameter to estimate the force potential of a muscle. Von Scheven [23] calculated the PCSA for the cranial middle and caudal LG and detected that the caudal part has significantly higher PCSA. Volumes, and therefore PCSA, vary widely in our study because of the different races, ages and sizes of the horses. In addition, using formalin fixation leads to tissue shrinkage and confounds the interpretation of total measures [19]. Therefore, we decided to leave measures of volume and calculation of PCSA as important parameters of muscle architecture in the results section, but do not use them for discussion to avoid overinterpretation of heterogenous samples and methodological influence on total measures and, consequently, this part of the hypothesis could neither be supported nor rejected.

Muscle function is not only determined by its inner architecture, but partly also by its muscle fibre type composition. Muscles can be more postural or locomotory, depending on if they have more slow type I muscle fibres or more fast type II muscle fibres [36,37,38,39].

Previous studies showed that most muscles in horses have a combination of different muscle fibre types, but fast type 2 muscle fibres are usually more common [37,40]. We exemplarily also took fresh muscle samples of superficial and deep aspects of six different positions along the thoracic and lumbar longissimus muscle to underline fibre type results for horses that have already been published [37,40]. Together with the fascicle data, the information on fibre type and diameter allows a broader view on the physiological parameters of the muscle. All of our samples showed relatively more fast type II (72.81% ± 6.07) fibres and, therefore, our results match with the literature about the longissimus muscle [37,40] concerning muscle fibre type proportion. 

Studies on the gluteus medius muscle in horses [41] reported differences in fibre type composition in different sampling depths. An increasing number of slow type I fibres was found from superficial to deep. In the LG, we could not find differences concerning fibre type proportions between deep and superficial probes. Kawai et al. [40] found regional differences in fibre type proportion between the thoracic und lumbar LG. Similarly to others [8], we could not confirm differences in fibre type proportion at the sampling sites along the longissimus muscle. 

In our muscle samples, the measured mean muscle fibre diameter of the fast type II fibres seemed to be slightly larger than the slow type I fibres, although we did not perform any statistical analysis due to small sample size. These results match with other studies about the equine gluteus muscle [41] and the equine lumbar longissimus muscle [42].

For both fibre type proportion and muscle fibre diameter, it has been demonstrated that the age and training status of the horse has an impact [43]. Further studies will be needed to confirm these findings as we only had samples from one muscle to examine fibre size and fibre type proportion.

## 5. Conclusions

**Figure 10:**
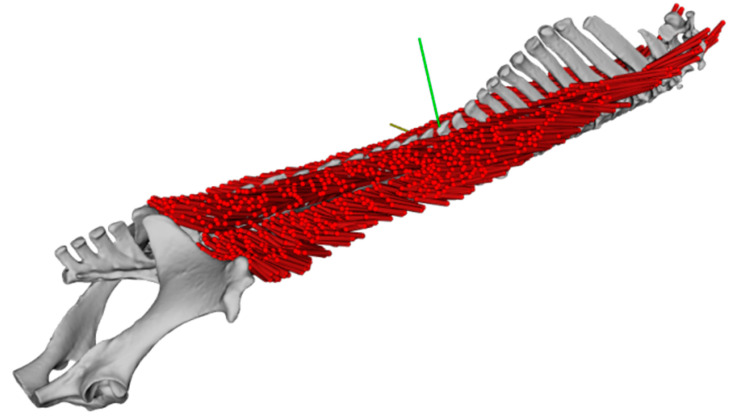
The model of the longissimus dorsi muscle based on the fibre digitalisation of the study. The model was used to simulate muscle activity within OpenSim software.

Anatomical observations and three-dimensional measurements reveal a very complex inner structure of the LG in the horse. Based on our three-dimensional model, the thoracolumbar LG of the horse with its multiple origins and areas of insertion and its complex inner structure due to the inner fascia could be described, and are also shown in a three-dimensional model for the first time. Muscle fascicle architecture changes within the LG from cranial to caudal. These regional anatomical differences indicate that the LG performs different functions along its length. Data for fascicle architecture such as fascicle length and fascicle orientation using the digitisation tools make all the measurements visible and represent the muscle fascicle architecture in its entirety. With the obtained detailed data, a base for enhanced understanding and further biomechanical studies, such as simulating the muscle function, is laid (see Figure 10).

## Data Availability

Data are available at the University of Veterinary Medicine Vienna. **Acknowlegements**: Open Access Funding by the Austrian Science Fund (FWF).

## References

[1] Stubbs N.C., Hodges P.W., Jeffcott L.B., Cowin G., Hodgson D.R., McGowan C.M. (2006). Functional anatomy of the caudal thoracolumbar and lumbosacral spine in the horse. Equine Vet. J..

[2] Jeffcott L.B. (1980). Disorders of the thoracolumbar spine of the horse—A survey of 443 cases. Equine Vet. J..

[3] Haussler K.K., Erb H.N. (2006). Pressure algometry for the detection of induced back pain in horses: A preliminary study. Equine Vet. J..

[4] Jeffcott L.B. (1999). Historical perspective and clinical indications. Vet. Clin. N. Am. Equine Pract..

[5] Pongratz U., Licka T. (2017). Algometry to measure pain threshold in the horse’s back—An in vivo and in vitro study. BMC Vet. Res..

[6] Wakeling J.M., Barnett K., Price S., Nankervis K. (2006). Effects of manipulative therapy on the longissimus dorsi in the equine back. Equine Comp. Exerc. Physiol..

[7] Haussler K.K. (1999). Anatomy of the thoracolumbar vertebral region. Vet. Clin. N. Am. Equine Pract..

[8] Ritruechai P., Weller R., Wakeling J.M. (2008). Regionalisation of the muscle fascicle architecture in the equine longissimus dorsi muscle. Equine Vet. J..

[9] Chiou W.K., Wong M.K., Lee Y.H. (1994). Epidemiology of low back pain in Chinese nurses. Int. J. Nurs. Stud..

[10] Lieber R.L., Friden J. (2000). Functional and clinical significance of skeletal muscle architecture. Muscle Nerve.

[11] Burkholder T.J., Fingado B., Baron S., Lieber R.L. (1994). Relationship between muscle fiber types and sizes and muscle architectural properties in the mouse hindlimb. J. Morphol..

[12] Gans C. (1982). Fiber architecture and muscle function. Exerc. Sport Sci. Rev..

[13] Neufuss J., Hesse B., Thorpe S.K.S., Vereecke E.E., D’Aout K., Fischer M.S., Schilling N. (2014). Fibre type composition in the lumbar perivertebral muscles of primates: Implications for the evolution of orthogrady in hominoids. J. Anat..

[14] Sacks R.D., Roy R.R. (1982). Architecture of the Hind Limb Muscles of Cats: Functional Significance. J. Morphol..

[15] Stark H., Fröber R., Schilling N. (2013). Intramuscular architecture of the autochthonous back muscles in humans. J. Anat..

[16] Licka T., Frey A., Peham C. (2009). Electromyographic activity of the longissimus dorsi muscle in horses when walking on a treadmill. Vet. J..

[17] Peham C., Schobesberger H. (2006). A novel method to estimate the stiffness of the equine back. J. Biomech..

[18] Robert C., Audigie F., Valette J.P., Pourcelot P., Denoix J.M. (2001). Effects of treadmill speed on the mechanics of the back in trotting saddlehorse. Equine Vet. J..

[19] Kikuchi Y., Kuraoka A. (2014). Differences in the muscle dimensional parameters between non-formalin-fixed (freeze-thawed) and formalin-fixed specimen in gorilla (Gorilla gorilla). Mammal Study.

[20] Gerling M.E., Brown S.H.M. (2013). Architectural analysis and predicted functional capability of the human latissimus dorsi muscle. J. Anat..

[21] Kim S.Y., Boynton E.L., Ravichandiran K., Fung L.Y., Bleakney R., Agur A.M. (2007). Three- Dimensional Study of the Musculotendinous Architecture of Supraspinatus and Its Functional Correlations. Clin. Anat..

[22] Lee D., Ravichandiran K., Jackson K., Fiume A.A. (2012). Robust estimation of PCSA and geometric reconstruction for human skeletal muscle. J. Biomech..

[23] Von Sheven C.C.A. (2010). The Anatomy and Function of the Equine Thoracolumbar Longissimus Dorsi Muscle. Ph.D. Thesis.

[24] Chow R.S., Medri M.K., Martin D.C., Leekam R.N., Agur A.M., McKee N.H. (2000). Sonographic studies of human soleus and gastrocnemius muscle architecture: Gender variability. Eur. J. Appl. Physiol..

[25] Crook T.C., Cruickshank S.E., McGowan C.M., Stubbs N., Wakeling J.M., Wilson A.M., Payne R.C. (2008). Comparative anatomy and muscle architecture of selected hind limb muscles in the Quarter Horse and Arab. J. Anat..

[26] Wichiewicz T.L., Roy R.R., Powell P.L., Perrine J.J., Edgerton V.R. (1984). Muscle architecture and force-velocity relationships in humans. J. Appl. Physiol. Respir. Environ. Exerc. Physiol..

[27] Townsend H.G.G., Leach D.H. (1984). Relationship between intervertebral joint morphology and mobility in the equine thoracolumbar spine. Equine Vet. J..

[28] Denoix J.M. (1999). Spinal biomechanics and functional anatomy. Vet. Clin. N. Am. Equine Pract..

[29] Bojadsen T.W.A., Silva E.S., Rodrigues A.J., Amadio A.C. (2000). Comparative study of Mm. Multifidi in lumbar and thoracic spine. J. Electromyogr. Kinesiol..

[30] Gans C., Bock W.J. (1965). The functional significance of muscle architecture: A theoretical analysis. Ergeb. Anat. Entwicklungsgesch..

[31] Faber M., Johnston C., Schamhardt H., van Weeren R., Roepstorff L., Barneveld A. (2001). Basic three-dimensional kinematics of the vertebral column of horses trotting on a treadmill. Am. J. Vet. Res..

[32] Jeffcott L.B., Dalin G. (1980). Natural rigidity of the horse’s backbone. Equine Vet. J..

[33] Maganaris N.C., Baltzopoulos V., Sargeant A.J. (1998). In vivo measurements of the triceps surae complex architecture in man: Implications for muscle function. J. Physiol..

[34] Felder A., Ward S.R., Lieber R.L. (2005). Sarcomere length measurement permits high resolution normalization of muscle fiber length in architectural studies. J. Exp. Biol..

[35] Powell P.L., Roy R.R., Kanim P., Bello A.M., Edgerton V.R. (1984). Predictability of skeletal muscle tension from architectural determinations in guinea pig hindlimbs. J. Appl. Physiol..

[36] Boyd-Clark L.C., Briggs C.A., Galea M.P. (2001). Comparative histochemical composition of muscle fibers in a pre- and a postvertebral muscle of the cervical spine. J. Anat..

[37] Hyytiäinen H.K., Mykkänen A.K., Hielm-Björkman A.K., Stubbs N.C., McGowan C. (2014). Muscle fibre type distribution of the thoracolumbar and hindlimb regions of horses: Relating fibre type and functional role. Acta Vet. Scand..

[38] MacDonald D.A., Moseley G.L., Hodges P.W. (2006). The lumbar multifidus: Does the evidence support clinical beliefs?. Man. Ther..

[39] Mannion A.F., Dumas G.A., Cooper R.G., Espinosa F.J., Faris M.W., Stevenson J.M. (1997). Muscle fibre size and type distribution in thoracic and lumbar regions of erector spinae in healthy subjects without low back pain: Normal values and sex differences. J. Anat..

[40] Kawai M., Minami Y., Sayama Y., Kuwano A., Hiraga A., Miyata H. (2009). Muscle fiber population and biochemical properties of whole body muscles in Thoroughbred horses. Anat. Rec..

[41] Rivero J.L.L., Ruz M.C., Serrano A.L., Diz A.M. (1995). Effects of a 3 month endurance training programme on skeletal muscle histochemistry in Andalusian, Arabian and Anglo-Arabian horses. Equine Vet. J..

[42] Ravara B., Gobbo V., Carraro U., Gelbmann L., Pribyl J., Schils S. (2015). Functional electrical stimulation as a safe and effective treatment for equine epaxial muscle spasms: Clinical evaluations and histochemical morphometry of mitochondria in muscle biopsies. Eur. J. Transl. Myol..

[43] Rivero J.L.L., Galisteo A.M., Agüera E., Miro F. (1993). Skeletal muscle histochemistry in male and female Andalusian and Arabian horses of different ages. Res. Vet. Sci..

